# Rodent Papillomaviruses

**DOI:** 10.3390/v9120362

**Published:** 2017-11-27

**Authors:** Aayushi Uberoi, Paul F. Lambert

**Affiliations:** McArdle Laboratory for Cancer Research, Department of Oncology, University of Wisconsin, Madison, WI 53705, USA; aayushi.uberoi@wisc.edu

**Keywords:** papillomaviruses, preclinical models, rodent papillomaviruses, murine papillomavirus

## Abstract

Preclinical infection model systems are extremely valuable tools to aid in our understanding of Human Papillomavirus (HPV) biology, disease progression, prevention, and treatments. In this context, rodent papillomaviruses and their respective infection models are useful tools but remain underutilized resources in the field of papillomavirus biology. Two rodent papillomaviruses, MnPV1, which infects the *Mastomys* species of multimammate rats, and MmuPV1, which infects laboratory mice, are currently the most studied rodent PVs. Both of these viruses cause malignancy in the skin and can provide attractive infection models to study the lesser understood cutaneous papillomaviruses that have been frequently associated with HPV-related skin cancers. Of these, MmuPV1 is the first reported rodent papillomavirus that can naturally infect the laboratory strain of mice. MmuPV1 is an attractive model virus to study papillomavirus pathogenesis because of the ubiquitous availability of lab mice and the fact that this mouse species is genetically modifiable. In this review, we have summarized the knowledge we have gained about PV biology from the study of rodent papillomaviruses and point out the remaining gaps that can provide new research opportunities.

## 1. Introduction

“Our attention was recently called to a disease occurring in wild cottontail rabbits in northwestern Iowa. Rabbits shot there by hunters were said to have numerous horn-like protuberances on the skin over various parts of their bodies. The animals were referred to popularly as “horned” or “warty” rabbits”.

Shope and Hurst [1].

In 1933, Dr. Richard Shope reported the discovery of a virus that could cause papillomas in cottontail rabbits [1]. This virus, which we know as the Shope papillomavirus or cottontail rabbit papillomavirus, was the first to etiologically associate a DNA virus with malignant progression in a mammalian species. A half century later, Dr. Harald zur Hausen discovered that human papillomaviruses (HPV16 and HPV18) [2,3] cause cervical cancer [4]. His Nobel Prize winning research provided momentum to the field of papillomavirus research, and since then many significant advances have been made in the field of papillomavirus tumor biology.

Papillomaviruses are non-enveloped, double-stranded DNA viruses that can infect mucosal and/or cutaneous epithelia, and are largely species specific. As of today, 230 types of human papillomaviruses and 159 types of non-human papillomaviruses have been identified (https://pave.niaid.nih.gov/ [5]). Mucosotropic human papillomavirus (HPVs) are the most common sexually transmitted pathogens, and a subset of these viruses cause 5% of human cancers, including cervical cancer, other anogenital cancers, and a growing fraction of head and neck cancers [6]. Other HPVs cause cutaneous warts, which are among the most common ailments that are treated by dermatologists [7,8,9,10]. They arise most frequently in children [11,12] and impose a significant burden to immunocompromised patients, particularly amongst organ transplant recipients [13,14,15]. They are ubiquitous in nature and can persist in the skin asymptomatically for years [13,16]. A subset of cutaneous HPVs also has been causally associated with skin cancer [13,17,18].

When considering that papillomaviruses are species-specific, and their life cycle is dependent on epithelial differentiation, no animal model of human papillomavirus (HPV) infection exists. Although organotypic raft cultures have greatly aided our understanding the life cycles of high-risk mucosal HPVs [19], papillomavirus researchers have had to rely on animal papillomaviruses to study viral pathogenesis, virus-host interactions, and immune responses to PVs. Initial studies using animal papillomaviruses tropic to cattle, rabbits, or dogs led to significant advances in our basic understanding of papillomavirus biology. From these studies, we gained fundamental knowledge about viral gene function, tissue tropism, cancer progression, vaccine efficacy, and therapeutics. Recent reviews describe the updates made in these classically used animal models [20,21,22,23,24,25,26]. Among these, bovine papillomavirus type 1 (BPV1) was the first papillomavirus genome to be sequenced [27]. BPV1 produces fibropapillomas on cattle, causes tumors in rodents and transforms fibroblasts in culture [1,28,29]. BPV1 and other BPV genotypes have been used to study both cutaneous and mucosal infections and PV-associated cancers. The ability of BPV1 to establish a nonproductive infectious state in rodent cells in tissue culture, and to morphologically transform these cells, drove early studies in the 1980s that helped to define the molecular genetics of papillomaviruses. Of particular note was the discovery of the *E5* oncogene and elucidation of its ability to activate the platelet derived growth factor receptor [30,31,32,33]. The revelation in the mid-1980s that certain HPVs could cause human cancer, and that these “high-risk” HPVs have the ability to immortalize human keratinocytes led investigators largely to abandon BPV1 as a model system by the early 1990s.

Canine oral papillomavirus (CPV1) was the first canine PV to be studied [34,35] and is highly relevant to HPVs that cause anogenital and head and neck cancers due to the mucosotropic nature of this virus. This model was valuable in assessing the efficiency of virus-like particle (VLP)-based and DNA-based prophylactic vaccines [36,37,38,39]. As described earlier in this review, experiments with the cutaneous cottontail rabbit papillomavirus (CRPV1) that causes skin malignancy, began in the 1930s [1]. Further studies demonstrated that various carcinogenic compounds could accelerate malignant progression rates [40] and provided an important preview into the subsequent connection between several high-risk HPV types and cervical cancer [22,41,42,43,44,45,46]. These classically studied preclinical models continue to offer valuable opportunities to study fundamental properties of papillomaviruses, such as tissue restriction and the function of viral genes in the context of intact hosts with functional immune systems.

As one can appreciate, while these models have been invaluable in educating us about PV biology, our understanding of papillomavirus biology remains limited, as these are not tractable laboratory animal models and the host species cannot be easily manipulated genetically, thus limiting mechanistic studies. A naturally occurring PV infection in rodent species, specifically laboratory mice, would provide not only a major new means to investigate the molecular pathogenesis of this group of viruses, but also the means to study the biology of the host responses and to identify factors that influence susceptibility to papillomavirus infections. In the last several decades, a dozen papillomaviruses that infect various rodent species have been isolated (Table 1), paving the way to such opportunities.

Advances in cloning methods have facilitated the discovery of rodent papillomaviruses [60,61]. Some of these papillomaviruses were isolated from exophytic lesions that arose in animals in the wild, such as the North American porcupine and beaver, Syrian golden hamster, and most recently, the Siberian hamster [48,49,50,58]. Others were cloned from skin swabs obtained from rodents including two novel species of rat papillomaviruses (RnPV2, 3) and one from the field mouse (AsPV1) [47]. The most extensively studied rodent papillomaviruses are MnPV1, which was the first rodent PV to be discovered (in 1984) and infects the multimammate rat, and MmuPV1, which was discovered in 2011, and is the first papillomavirus identified to naturally infect the laboratory strain of mouse, *Mus musculus* [54]. In the last six years, rapid progress has been made in understanding the biology of MmuPV1, which represents an attractive model to study papillomavirus pathogenesis due to the ubiquitous availability of laboratory mice and the fact that this mouse species is easily genetically modifiable [62]. Rodent PVs are a fledgling area of PV research and provide ample opportunities to understand PV biology. In this review, we have summarized the knowledge that we have gained about PV biology from the study of rodent papillomaviruses.

## 2. Genomic Analysis of Rodent Papillomaviruses

**Figure 1:**
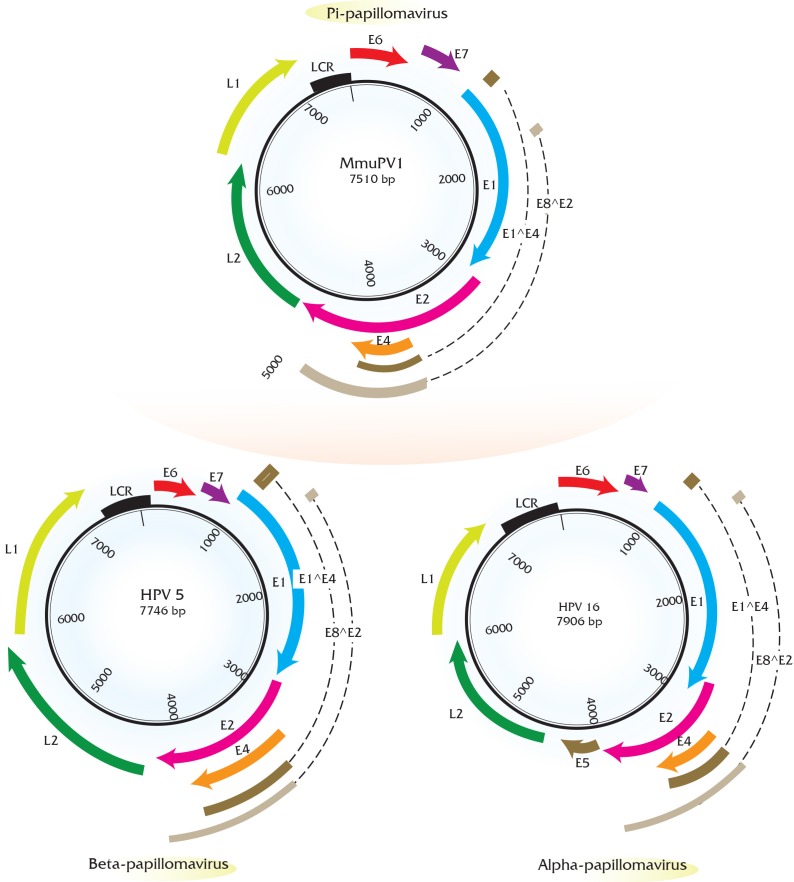
Genome organization of rodent papillomaviruses compared to high-risk papillomaviruses. Rodent PVs follow similar genome organization as prototype papillomaviruses and contain early and late open reading frames (ORFs). Shown here is the mouse papillomavirus (MmuPV1) genome (**top**) as an example of rodent papillomaviruses. For comparison, genomes of prototype high-risk HPVs i.e., HPV5 (**bottom left**, cutaneous Human Papillomavirus (HPV) as an example of beta-papillomaviruses) and HPV16 (**bottom right**, mucosal HPV as an example of alpha papillomaviruses) have been shown. The early ORF of MmuPV1 and HPV5 consists of *E1* (blue), *E2* (pink), *E4* (orange), *E6* (red) and *E7* (purple) and the late ORF consists of *L2* (green), *L1* (lime). At the genomic level, the most notable change in rodent PV organization compared to HPV16 is the lack of *E5* oncogene (brown), which is present in HPV16. Genes *E5*, *E6*, *E7* have been shown to be oncogenic in the context of several papillomaviruses. The long control region (LCR) is shown in black. *E1^E4* (dark brown) and *E8^E2* (light brown) splice products are also indicated based on transcript maps of these viruses.

Papillomaviruses consist of a double-stranded, circular genome of around 8 kb containing one coding strand that is encapsidated in an icosahedral protein shell made up of a major (L1) and minor (L2) capsid protein [63]. Rodent PV DNA genomes follow the same general organization of genetic elements, as seen in prototype papillomaviruses (e.g., HPV16 and BPV1) [64,65,66]. Approximately half of the viral genome contains translational open reading frames (ORFs) that encode for the so called “early” or *E* gene products. These early gene products are responsible for viral replication and maintenance, interact with multiple host factors to facilitate the viral life cycle, and contribute to the neoplastic properties of these DNA tumor viruses. The “late” or *L* ORFs encode for the proteins that form the viral capsid (Figure 1).

**Figure 2:**
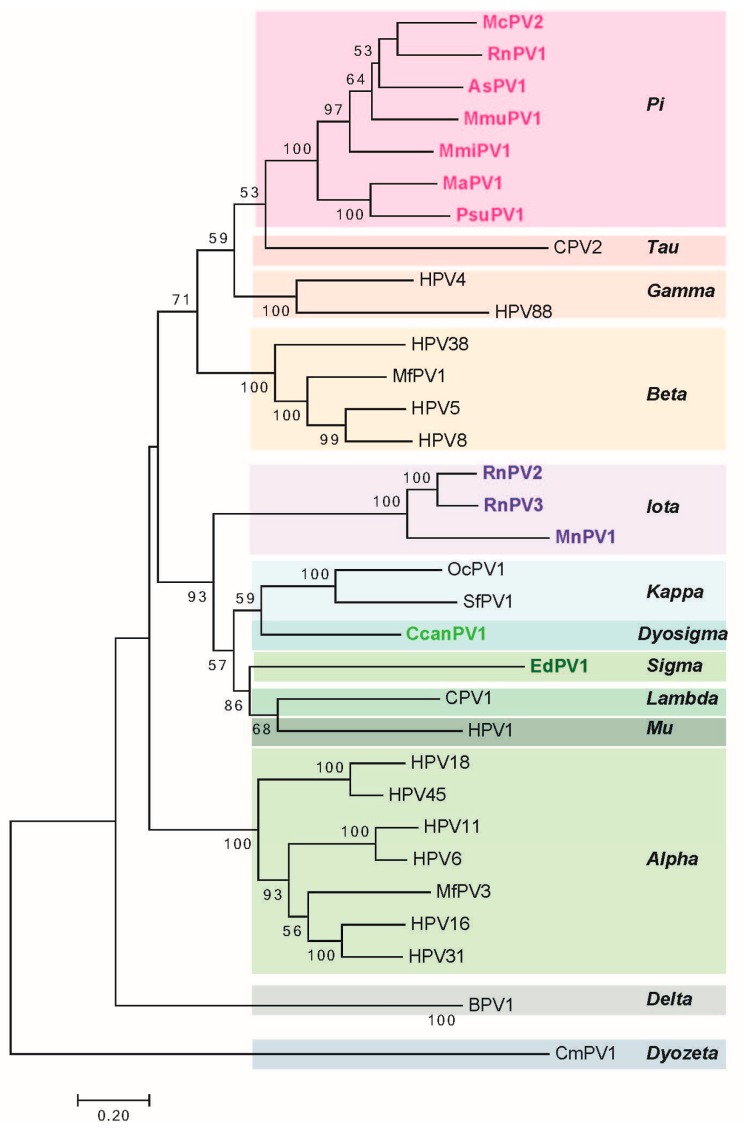
Phylogenetic analysis of rodent PVs. Phylogenetic tree construction of rodent PVs was performed on the basis of *L1* nucleotide sequences as per the standard criteria previously set for PV classification [72]. The evolutionary history was inferred by using the Maximum Likelihood method based on the General Time Reversible model, and the tree with the highest log likelihood (−31,759.0931) is shown. The percentage of trees in which the associated taxa clustered together is shown next to the branches. Values less than 50% are not shown. Initial tree for the heuristic search was obtained automatically by applying Neighbor-Join and BioNJ algorithms to a matrix of pairwise distances estimated using the Maximum Composite Likelihood (MCL) approach and then selecting the topology with superior log likelihood value. A discrete Gamma distribution was used to model evolutionary rate differences among sites (5 categories (+G, parameter = 0.9374)). The rate variation model allowed for some sites to be evolutionarily invariable (9.1152% sites). The tree is drawn to scale with branch lengths measured in the number of substitutions per site. All positions containing gaps and missing data were eliminated. There were a total of 1311 positions in the final dataset. Evolutionary analyses were conducted in MEGA7 (Version 7.0.21, Pensylvania State University, Hershey, PA, USA) [73]. In this small-scale phylogenetic analysis 33 papillomaviruses that represent prototype papillomaviruses in each clade were chosen for the analysis. For the most recent complete phylogenetic analysis of all papillomavirus species, the reader is referred to de Villiers, (2013) [74].

The long control region (LCR, also referred to as the upstream regulatory region (URR) or untranslated region (UTR)) carries cis elements required for DNA replication (i.e., the viral origin of DNA replication), as well as multiple transcriptional promoters and transcriptional enhancer elements. Phylogenetic analysis of rodent PVs based on *L1* nucleotide sequence by maximum likelihood estimation reveals that rodent PVs are not monophyletic (Figure 2) consistent with previous reports [47,59]. They are found at disparate positions on the phylogenetic tree, in the Pi, Iota, Sigma, or Dyo-sigma lineages [47]. This observation provides valuable insight into the evolution of papillomaviruses. It has long been hypothesized that papillomaviruses co-evolved with their hosts [67]. Were this hypothesis correct, then all of the PVs infecting the same host would appear close together on the phylogenetic tree because they would have shared a recent common ancestor [68,69]. However, the existence of distantly related rodent PV lineages is inconsistent with the predictions of this hypothesis [47,70]. For instance, different PV types, each isolated from *Rattus norvegicus* (RnPV1-3) and *Mastomys coucha* (MnPV1 and McPV2) do not constitute monophyletic groups. RnPV1 belongs to Pi-PVs, whereas RnPV2 and RnPV3 belong to the distant iota-PVs. Likewise is true for *Mastomys*-associated PVs where McPV2 is a Pi-PV and MnPV1 is an iota-PV. Generally, viral tropism is conserved among the different PV lineages. For example, Alpha-PVs largely comprise of mucosotropic papillomaviruses with the exception of the cutaneotropic HPV2 and HPV4 [71]. While the tropism of most rodent PVs remains under investigation, at least Pi-PVs are comprised of both cutaneotropic and mucosotropic types of viruses. For example, MmiPV1 is associated with skin infections in *Micromys minutis*, whereas, McPV2 is associated with genital infections in *Mastomys coucha.* Mouse papillomavirus (MmuPV1) has been suggested to have an expanded tropism in its host species as *Mus musculus* is reported to induce disease in both cutaneous and mucosal epithelium [62].

The early region of all the rodent PVs consists of five translational open reading frames (ORFs): *E1*, *E2*, *E4*, *E6*, and *E7* (Figure 1). Among these, the *E1* and *E2* ORFs encode factors that are key for viral DNA replication and transcription similar to other papillomaviruses [75,76]. Rodent papillomaviruses possess a clearly recognizable *E4* ORF that is present in most other papillomaviruses except for avian papillomaviruses [77]. Three papillomavirus early gene products, *E5*, *E6*, and *E7*, have been shown to stimulate cell proliferation and survival and modulate keratinocyte differentiation; therefore, these gene products are considered to be oncoproteins in the case of PVs associated with cancer. Beta papillomaviruses comprising largely of cutaneotropic HPVs lack an *E5* ORF, which is hypothesized to contribute to their inability to cause cancers. This is in contrast to the alpha-viruses, including the high-risk mucosotropic papillomaviruses that do contain E5 [68,78]. Similar to beta-HPVs, all of the rodent PVs lack an *E5* ORF, but possess the *E6* and *E7* ORFs. Great efforts have been made in the field to elucidate the oncogenic roles of *E6* and *E7* encoded by high-risk mucosotropic HPVs [79,80]. The biological activities of rodent PV *E6* and *E7* gene products remain under investigation and are described in Section 5.

## 3. Pathogenesis by Rodent Papillomaviruses

### 3.1. Mastomys-Associated Papillomaviruses

Studies in multimammate mice belonging to the *Mastomys* genus of the rodent family *Muridae* have provided valuable insights into the biology of papillomaviruses. The first-discovered rodent papillomavirus was isolated from skin keratoacanthomas arising spontaneously in a colony of African multimammate rats, *Mastomys coucha* [81,82,83,84,85], originally misidentified as *Mastomys natalensis* [86], leading to its designation as the *Mastomys natalensis* papillomavirus (MnPV1). *M. natalensis* (common name: Natal multimammate mouse) and *M. coucha* (common name: Southern multimammate mouse) differ karyotypically, but are difficult to distinguish visually, leading to confusion in their identity.

Studies pertaining to these papillomaviruses have been performed in a *M. coucha* colony maintained at the German Cancer Research Center (DKFZ), in which animals were found to be naturally and persistently infected with two rodent papillomaviruses—the aforementioned *Mastomys natalensis* papillomavirus (MnPV1) and a second virus called *Mastomys coucha* papillomavirus-2 (McPV2) [51]. Animals in this laboratory colony have been found to be latently infected with MnPV1 and to a lesser extent with McPV2 as determined by detection of viral DNA, as well as by serological studies [87]. Recently, a virus-free colony of the *M. coucha* was established at the DFKZ [88]. Of the two *Mastomys*-associated papillomaviruses, McPV2 was only recently isolated from anogenital condylomas in aged *M. coucha* mice [51], and consequently, its biology is less well understood. However, substantial efforts have been made in understanding biology of MnPV1, which is associated predominantly with cutaneous lesions.

MnPV1-associated skin lesions develop spontaneously on exposed surfaces of *M. coucha*. These lesions present as exophytic papillomas, are highly keratinized, and do not regress [81]. The MnPV1 DNA genome exists as extrachromosomal circular DNA in the skin lesions [57]. While only 3% of young animals spontaneously develop overt MnPV1-associated lesions, the frequency increases to 30–40% of animals at one year of age [57]. MnPV1-DNA based in situ hybridization analysis of skin papillomas from infected animals showed the presence of amplified viral DNA predominantly in subrabasal epithelial layers [89]. MnPV1 genomes were also detected in the dermal papilla of the hair follicles [89], which is a known reservoir of stem cells [90]. This is an intriguing observation as previous studies with CRPV in rabbits found viral gene expression in hair follicles, raising the possibility that hair follicle stem cells might be a target for papillomavirus infections [91]. The MnPV1 infection model also appears to represent an intriguing case of “endogenous infection” as extrachromosomal DNA is found to occur in other internal tissues (e.g., liver, brain and lungs) in both tumor-free and tumor-bearing animals [57,89]. Nafz, et al. (2007) reported that fetuses or newborns from MnPV1-infected pregnant dams did not show any signs of MnPV1 infection suggesting that infection occurs after birth [89]. In the same study, the authors reported that two out of six tumor-free animals showed the presence of MnPV1 DNA in their blood. While analysis of more samples is required, this observation suggests that MnPV1 spread could occur via blood.

MnPV1 also has shed light on the concept of papillomaviral latency, which is described as a phase of the viral life cycle in which “no clinical signs of disease are apparent and new virus particles are not produced and released” [92]. MnPV1 can be found to persist in young animals at low viral copy numbers without any overt sign of disease, as consistent with latency. Upon chronic topical administration of the tumor promoter, 12-*O*-Tetradecanoylphorbol-13-acetate (TPA) to the skin of such mice, an earlier onset of MnPV1-induced papillomas was observed that contained higher viral DNA loads [57]. Mechanical irritation of animals latently infected with MnPV1 also led to an earlier onset of keratoacanthomas and increased viral DNA loads [93]. These observations indicate that the disruption of epidermal homeostasis triggers latent papillomavirus to establish a productive infection that results in overt clinical disease [93].

While MnPV1 mostly causes benign lesions, interesting insights have been gained about the role of MnPV1 in carcinogenesis in conjunction with chemical agents. Chronic administration of the chemical carcinogen, 7,12-dimethylbenz(a)anthracene (DMBA), led to both benign and malignant tumors in all animals. Furthermore, in a classical two-stage carcinogenesis study, MnPV1 led to squamous cell carcinomas (SCC) after a single topical application of DMBA, followed by repeated challenge with TPA [94]. To understand the biology of the MnPV1 putative oncogene, *E6*, transgenic mice expressing the MnPV1 *E6* gene in the skin under the control of the human cytokeratin-14 promoter were generated. MnPV1-*E6* transgenic mice do not develop papillomas or keratoacanthomas spontaneously. However, squamous cell carcinomas were observed in MnPV1-*E6* mice in conjunction with classical two-stage skin carcinogenesis studies, as described earlier, indicating that MnPV1 *E6* can cooperate with chemical carcinogens to cause cancer [95]. To date, this is the only transgenic mouse model expressing a putative rodent papillomavirus oncogene in the epithelia. At present, the biological interaction partners of MnPV1 *E6* remain largely unknown as a lack of reagent availability coupled with the lack of in vitro systems has impeded progress in this area. In this context the establishment of the first ever keratinocyte cell line from *M. coucha* provides a potential cell culture system to study the virus life cycle and interaction partners in vitro [96]. Also, information derived from the recent transcriptome analysis of MnPV1 using high-throughput RNAseq analyses of productive lesions led to the assembly of a comprehensive transcript map with the identification of several novel splicing patterns for MnPV1 (see Section 5). This knowledge will be valuable in moving forward the study of MnPV1 molecular biology [97].

### 3.2. Mus Musculus-Associated Papillomaviruses

#### 3.2.1. Discovery of the First Papillomavirus to Infect the Standard Laboratory Mouse Strain, *Mus musculus*

MmuPV1 is the first cloned rodent papillomavirus to be isolated from the laboratory strain of mice, *Mus musculus* [54]. Subsequently, a variant of the MmuPV-1 with very high sequence similarity (99%) was independently isolated from the skin of a house mouse [47]. MmuPV1 is a valuable papillomavirus as it allows us the opportunity to study papillomavirus infections in a natural setting in a genetically modifiable organism. MmuPV1 was isolated from florid papillomas that arose spontaneously around the muzzle and nose regions in a colony of T-cell deficient NMRI-*FoxN1^nu^*^/*nu*^ strain of mice at the Advanced Centre for Treatment and Research in Cancer (ACTREC) in India [54]. Histopathological analysis of these lesions showed features that were consistent with papillomatosis i.e., papillary projections with koilocytes that were positive for PV group-specific antigens. The viral extracts from these papillomas were found to be transmissible to other NMRI-*FoxN1^nu^*^/*nu*^ mice. Lateral transmission of papillomavirus to uninfected sites was also observed in these animals [54,98,99], with naïve nude mice housed with infected nude mice developing MmuPV1-associated papillomas [99]. Lateral transmission of virus resulting in papillomas at uninfected sites was attributed to general grooming behavior of mice, which can lead to microabrasions eventually promoting infection by MmuPV1. Analysis of MmuPV1-induced lesions arising on the NCR (National Cancer Institute) strain of *FoxN1^nu^*^/*nu*^ suggests that this papillomavirus has an unusual pattern of late protein expression. In this study, the authors generated polyclonal antisera specific for L1 and L2 and observed that L1 protein was expressed in all the layers of the stratified epithelia of the papillomas, whereas L2 protein was detected only in the subrabasal compartments [98]. They also observed, unexpectedly, that the L1 expression was localized to the cytoplasm in lower layers of the epithelia prior to expression of L2. This is different from infections that are induced by high-risk HPVs, where neither L1 nor L2 are detected in lower layers of the epithelia and L2 expression is turned on in layers prior to initiation of L1 expression [100]. However, in cutaneous warts induced by HPV1, L1 is expressed throughout the epithelium and in lower layers than is detected L2 [101], similar to that seen with MmuPV1.

**Figure 3:**
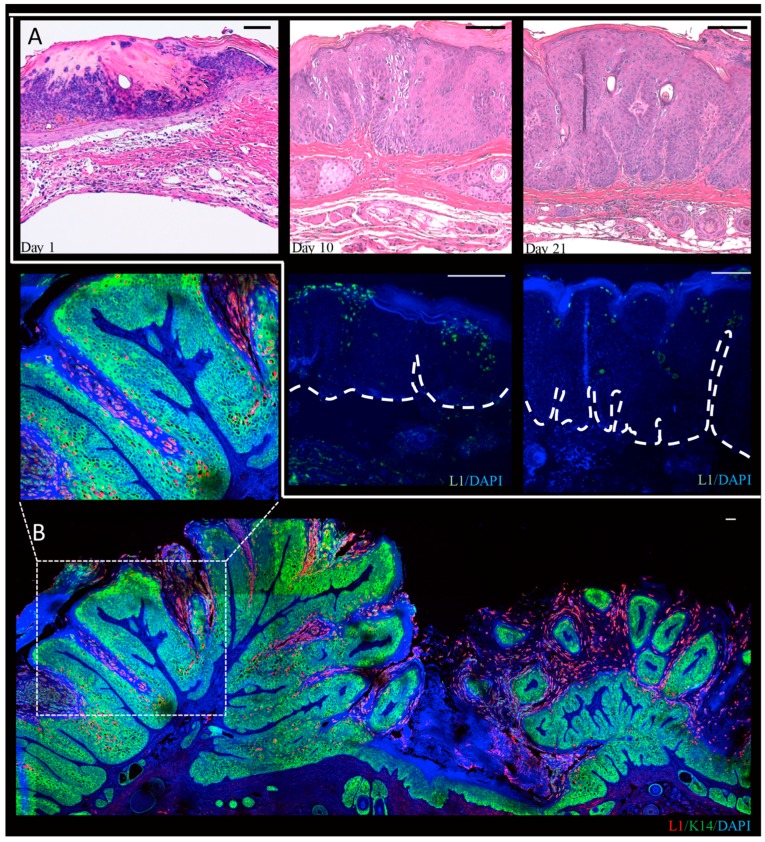
Progression of MmuPV1-induced tail papillomas in BALB/C-*FoxN1^nu^* nude mice. This figure has been adapted and modified from Xue, et al. (2017, in press) [102]. Nude mice were infected with MmuPV1 virus extract following scarification and tissue was harvested at days 1, 10 and 21 post-infection. **Panel A** shows H&E staining of infected tissue at indicated time points post-infection with MmuPV1 and the corresponding L1 immunofluorescence (green) to show presence of MmuPV1 capsid proteins. At day 1 we see presence of a scab and immune infiltration but no L1 was detected [102]. At day 10 post-infection we can see hyperplasia, and formation of fibrillary projections begin to appear indicating formation of papilloma. At this time point we can detect L1 in the suprabasal layers. At day 21, we can see more pronounced papillomatosis, and L1 staining can be seen even in basal layers of the papilloma. The H&E images and fluorescent images were captured using a Zeiss AxioImager M2 microscope and AxioVision software version 4.8.2 (ZEISS, Jena, Germany). **Panel B** shows a high resolution wide-field image of an MmuPV1-induced tail wart at 6 months post-infection. In this image we have shown L1-K14 dual immunofluorescence. Extensive papilloma formation coupled with expansion of the K14 layer (green) is seen, and L1 expression (red) is seen throughout the epithelia (inset). The nuclei are stained with DAPI (blue). High resolution wide-field fluorescent images were acquired by means of a super-resolution Leica SP8 STED confocal microscope (Leica Microsystems Inc., Buffalo Grove, IL, USA) equipped with a motorized stage located in the UW optical core. This microscope is equipped with PMT and HyD lasers. All of the images were taken by means of a 20× objective lens (Specifications: HC PL APO 20×/0.75 CS2, Dry). The images were acquired by tile-scanning by marking positions around the region of interest on the LAS-X suite (version: 2.0.1). The merged wide-field image was obtained by automatic stitching of individual styles by means of an in-built auto-stitching algorithm that is part of the LAS-X suite. Scale bar (100 μm) is shown in top right corner.

In our lab, we performed a time-course analysis, in which infected tissue was harvested three days, 10 days, and 21 days post-infection, and from mature warts at three or six months post-infection from MmuPV1-infected BALB/c-*FoxN1^nu^* mice (Figure 3) [102]. We observed that L1-positive cells are present throughout the epithelium in mature warts, but are restricted to more terminally differentiated cells at the earlier time points indicating that the full manifestations of the viral life cycle are realized at times later than four weeks post-infection. In these studies we noted that L1 expression was predominantly nuclear though some cytoplasmic signal was observed as well. This is consistent with recent observations that were made in NU/J-*FoxN1^nu^* mice [103]. Differences in the pattern of detection of L1 may reflect the different genetic backgrounds of the mice that were used in these studies. We also observed amplification of MmuPV1 DNA in the basal layer of the epithelia. Basal cells have been seen to support viral DNA amplification in lesions caused by the cutaneous HPV1 and HPV63 and by canine oral papillomavirus (COPV1), which is a mucosal papillomavirus [24,104]. We also observed that in experimentally infected nude mice that have developed warts at the sites of infection, other uninfected areas of the epidermis show evidence of subclinical infections (i.e., no signs of overt papillomas). At the microscopic level, these subclinical infections showed evidence for productive infection based upon the detection of viral DNA amplification and L1 expression [102]. This raises the intriguing possibility that subclinical PV infections may be common in immunodeficient or immunosuppressed contexts in humans as well. In this regard, organ transplant patients on immunosuppressants are known to have an increased abundance of HPV DNA in randomly sampled hair follicles from clinically normal skin [105].

L1 and L2 capsid proteins play key roles in in early events in PV infectious entry [106,107]. Studies attempting to tease apart early events in PV infectious entry have been performed using pseudoviruses (i.e., reporter plasmids encapsidated by PV L1 and L2) have produced different results, depending on the in vitro or in vivo model system utilized [106]. It is worth considering that several of these in vivo studies have been performed in the context of a heterologous host. Recently, it has been reported that murine skin, as well genital tissues, were similarly permissive to infection by pseudoviruses of multiples PV types that included HPV16 as well as MmuPV1 [108]. Despite this shared activity, pseudovirus infections performed in the context of cevicovaginal tract showed that HPV16 and MmuPV1 capsids interact with different host cell proteins [109]. Heparin sulfate proteoglycans (HSPGs) have been shown to be an initial attachment receptor for multiple PV genotypes in vitro [106,110], and have been demonstrated to contribute to infection via the binding of soluble complexes with growth factors [111]. It has been shown that the interaction of PVs with BM via HSPGs is critical for in vivo infection of mucosotropic HPV16 and HPV31, as well as the cutaneous HPV5. However, in the same study, it was demonstrated that different heparin variants affected the alpha and beta types in dissimilar ways raising the possibility that cutaneous and mucosal HPVs may have evolved to recognize different forms of HSPGs. Day, et al. (2015) demonstrated that unlike HPV16, MmuPV1 bound basement membrane (BM) in an HSPG-independent manner [109]. Despite the differences in the initial interacting partners found for HPV16 and MmuPV1, both of the viruses appear to use the same endocytic pathways, as is consistent with other HPV types [112]. It remains to be determined whether these interactions are identical for the cutaneous epithelia in the context of MmuPV1.

#### 3.2.2. T-Cell Deficient Strains are Susceptible to MmuPV1 Infection

Multiple other genetic backgrounds carrying the *FoxN1^nu^*^/*nu*^ mutation are also susceptible to infection by naked, recircularized MmuPV1 DNA, as well as quasivirions encapsidating synthesized MmuPV1 genomes (summarized in Table 2). Mice carrying the *FoxN1^nu^*^/*nu*^ mutation are null for the *FoxN1* (Forkhead Box N1) gene and are colloquially referred to as nude mice due to their characteristic hairless phenotype [113]. These mice are immunodeficient by virtue of thymic aplasia that results in T-cell deficiency [114]. MmuPV1 consistently causes exophytic papillomas in the muzzle and tail regions of nude mice, but torso skin appears to be resistant to MmuPV1 papillomatosis [98,99], except in the B6 and NMRI (Naval Medical Research Institute) genetic backgrounds where locally invasive trichoblastomas are observed on the torso [99]. This observation would suggest that the anatomical site of infection is a critical determinant of the nature of disease progression. Gene expression profiling studies performed recently on dorsal versus tail skin identified gene networks, including *Sonic Hedgehog (Shh), Wnt, Leucine-rich repeat containing G-protein coupled receptor (Lgr)* family of stem cell markers, and keratins that differed in expression at these tissue sites [115], providing a potential explanation for the differential susceptibility of dorsal and tail skin to the development of skin diseases and tumorigenesis. While titrating the virus dose to observe papilloma incidence in nude mice, we and others have found that the lowest dose of virus that can consistently cause papillomas at 100% of infected sites in nude mice is 10^7^ virus particles containing viral DNA [98,116]. In our long-term monitoring of papillomas with 10^6^ virus particles, we observed that there was a delay in onset of papillomatosis in nude mice with papilloma incidence increasing over time [116]. While MmuPV1 appears to be predominantly cutaneotropic, some groups have observed extended tropism of the virus in these immunodeficient strains. This is evidenced by the ability of the virus to cause lesions in anogential and vaginal areas, as well as in the oropharyngeal tract [117,118]. It has been shown that mice infected cutaneously display secondary infections at mucosal sites [118]. Recently, it has been reported that mice infected mucosally also display secondary infections at skin sites. Histopathological analysis of these skin lesions arising due to secondary infection in aged mice showed signs of micro-invasive squamous cell carcinoma [103].

Mice with a SCID (Severe combined immunodeficiency) mutation in the *Prkdc* (Protein Kinase, DNA-activated, Catalytic polypeptide) gene (*Prkdc^scid^*) on the SHO (SCID hairless outbred) genetic background are susceptible to MmuPV1 infection [121,122]. These mice are generally characterized by the absence of functional T cells and B cells, however they have been known to have leaky production of B cells as they age [123,124,125]. Interestingly, SCID mice on the NOD (non-obese diabetic) genetic background are resistant to MmuPV1-associated skin papillomas [99]. This observation suggests that genetic background is probably a key factor in determining the susceptibility to MmuPV1 infection. Lists of immunodeficient strains found to be susceptible or resistant to MmuPV1 infection are summarized in Table 2 and Table 3, respectively.

Multiple groups, including our lab, have reported that MmuPV1 does not cause papillomatosis in routinely used immunocompetent wild-type strains of mice, such as BALB/c, FVB/NJ, and C57/BL6 [54,55,98,99,116,120,126], although serological responses to MmuPV1 virus-like particles (VLPs) have been reported in C57/BL6 mice that are infected with MmuPV1 even 70 days post-infection [55]. If a very high dose of virus was used (1012 virus particles), papillomas did arise in C57/BL6 and SENCAR (SENsitivity to CARcinogenesis) strains of mice, but these papillomas regressed within one or two weeks post appearance [126]. Of these, the SENCAR strain of mice is a model that is classically used in skin tumorigenesis studies due to its high susceptibility to skin tumor induction by chemical carcinogens [127,128]. Continuous treatment with the immunosuppressant drug cyclosporine led to MmuPV1-induced papillomas in multiple immunocompetent mouse strains [120,126]. These observations have helped to define what immune defects lead to susceptibility to MmuPV1 papillomas. Genetically modified strains selectively deficient for B-cells or subpopulations of T-cells (CD4^+^, CD8^+^, or CD40^+^) did not develop MmuPV1-associated papillomas [99,120,126]. These data suggest that complete T-cell deficiency is required for MmuPV1 susceptibility, at least on the C57BL/6 genetic background. This conclusion was confirmed by performing antibody-based depletion studies using a monoclonal antibody against the CD3 T-cell co-receptor, which led to MmuPV1-induced papillomas in multiple strains [120,126]. It is notable that while in the SENCAR strain administration of antibodies against either CD4^+^ or CD8^+^ led to susceptibility to MmuPV1, in C57/BL6 and BALB/c genetic backgrounds, the elimination of both CD4^+^ and CD8^+^ cells was required. Studies that involved intervention with immunosuppressants have been summarized in Table 4. Recently, it has been reported that when MmuPV1-induced tumors arising in T-cell deficient mice were transplanted onto immunocompetent congenic mice, the tumors completely regressed [129]. Further, this elimination of MmuPV1-induced tumors was consistent with the induction of antitumor T cell immunity affirming the role of T-cell immunity in MmuPV1 infections.

CD8^+^ T-cells, specifically effector cytotoxic T lymphocytes (CTLs), play a critical role in the clearance of virally infected epithelial cells, as well as regression of epithelial neoplasia [130,131,132]. In several well-characterized systems, CD4^+^ T-cells are necessary for induction of primary CD8^+^ T-cell responses, as well as for their proliferation, activation, and differentiation into effector cytotoxic T lymphocytes. For example, in a murine model of herpes virus infection, CD4^+^ T cells were required for efficient local recruitment of herpes simplex virus-specific CD8^+^ T cells to the vaginal epithelium [133]. Therefore, the observation that loss of CD4^+^ T-cells in C57/BL6 and BALB/c was not sufficient to increase the susceptibility to MmuPV1-induced papillomas [99,120,126] is somewhat surprising. Furthermore, it was also observed that MmuPV1 infection sites in C57BL/6 mice had recruitment of CD8^+^ T-cells, even in the absence of CD4^+^ T-cells [126]. Recent studies have shown that CD4^+^ T helper-independent CD8^+^ CTL responses can be elicited by several other viruses as well (e.g., ectromelia virus [134], influenza virus [135], and dengue virus [136]). Although the details of immune recognition of MmuPV1 are yet to be determined, it seems likely that T-cells control MmuPV1 infection by having an effect on both innate and adaptive arms of the immune system. It is worth noting that increased an incidence of HPV-associated papillomas is observed in T-cell-immunosuppressed patients further emphasizing the role of CD4^+^ and/or CD8^+^ T-cell responses in PV-mediated disease [137]. T-cell infiltration is seen in both cutaneous and mucosal lesions during the spontaneous regression of papillomas [138,139,140,141]. When T-cell responses to five HPV16 proteins (E6, E7, E4, L1, and L2) were analyzed in women with CIN or cervical carcinoma, CD4^+^ and CD8^+^ T-cell responses changed with severity of disease, suggesting that there are differences in how viral antigens are seen by the immune system during progressive disease of the cervix [142]. Hence, there is a need to understand and define the role that is played by PV specific T-cells during the natural course of virus infection. While such a prospective study will be hard to conduct in humans, the MmuPV1-infection model provides us with the opportunity to define the contribution of T-cells during the course of PV infections.

While most of the studies in MmuPV1 have been focused on the role of the adaptive arm of the immune system, specifically T-cell biology, there have been limited efforts in looking at the role of innate immunity in this infection model as well. In a previous study, no visible disease was seen in cutaneous sites of Interferon-alpha/beta receptor alpha chain-null (*Ifnar^Ko^*) mice that lack Type I interferons [120]. However, in a recent review, it was shared that MmuPV1 DNA persisted longer in the anogenital and vaginal tracts of *Ifnar^Ko^* mice when compared to their wild-type counterparts [62]. It is worth appreciating that the immune biology of mucosal epithelia is very different from cutaneous epithelia and can contribute to progression of disease differently [143,144]. In this context, a role of natural killer (NK) cells has been proposed [62]; however, in earlier studies, NK-cell deficient mice were found to be resistant to MmuPV1 infection [126]. Overall, the reported studies indicate that T-cells primarily control phenotypic disease, which is caused by MmuPV1.

The MmuPV1-infection model affords us the opportunity to study the role of antiviral host-intrinsic immunity. Antiviral intrinsic immunity, a form of innate immunity that directly restricts viral replication and assembly, has been shown to be an important host-restriction factor in the context of influenza and retroviruses [145]. In the last decade, several host factors that restrict transcription (e.g., Sp100 nuclear antigen [146], Epidermodysplasia verruciformis proteins i.e. EVERs [147]) and early phases of viral replication (microRNA miR-145 [148], and interferon inducible proteins p56 [149] and IFI16 [150]) of papillomaviruses have also been characterized [151]. In recent years, the apolipoprotein B messenger RNA-editing, enzyme-catalytic, polypeptide-like 3 (APOBEC3) family of cytidine deaminases has emerged as a critical player in governing intrinsic immunity [152]. APOBEC3 enzyme APOBEC3A has been shown to cause mutations of HPV DNA [153] and inhibit HPV infectivity [154]. Furthermore in a large scale, case-control study of HPV16 associated cervical cancers and precancers, it was recently shown that HPV16 rare variants potentially induced by the antiviral activity of human APOBEC3 [155]. It has been reported that *Apobec3*^−/−^ mice (*n* = 3) were not susceptible to MmuPV1-induced tumors [154]. This preliminary study was performed in immunocompetent mice (that are not permissive to MmuPV1-induced papillomas) suggesting that perhaps the loss of APOBEC3 is not sufficient to overcome host adaptive immune responses against MmuPV1 infection in immunocompetent mice. The role of APOBEC3 in disease progression is yet to be determined.

#### 3.2.3. MmuPV1 Infection in Hairless Strains

In the original paper that described the discovery of MmuPV1, virus extracts from papillomas arising on nude mice were transmissible to other nude mice, as well as to the immunocompetent but hairless strain of *S/RV/Cri^ba^*^/*ba*^ mice [54]. Interestingly, while papillomas persisted in the originally infected NMRI-*FoxN1^nu^*^/*nu*^ strain that they completely regressed in *S/RV/Cri^ba^*^/*ba*^ mice 6–8 weeks post appearance. The *S/RV/Cri^ba^*^/*ba*^ is an outbred strain endemic to India [156,157,158,159], and is at least phenotypically similar to the outbred *SKH1-elite* strain in the United States that has an autosomal mutation in the *Hr^Hr^* (Hairless) allele resulting in a hairless phenotype. The *SKH1-elite* strain is a popular strain in the context of photocarcinogenesis studies [160], and subsequent studies by other groups have found that the *SKH1-elite* strain is also susceptible to MmuPV1-associated papillomas. In a recent, more comprehensive study, it was found that only a subset (22.5% i.e., 9/40 mice) of infected *SKH1-elite* mice developed papillomas, some of which (5/9 mice) persisted while others regressed (4/9) [120]. It is worth noting that the immunobiology of *SKH-1 elite* strain is poorly understood.

#### 3.2.4. Role of Ultraviolet Radiation (UVR) in MmuPV1-Associated Disease

There are multiple lines of evidence that suggest UVR could play a role in infection by cutaneous PVs. One line of evidence comes from epidemiological data that was gathered from human studies; and the other line of evidence comes from studying UVR in other animal models of PV infection. Epidemiological studies indicate there is a correlation between the exposure to ultraviolet radiation (UVR) from sunlight and the prevalence of cutaneous HPVs in healthy and immunosuppressed patients [161,162]. Cutaneous HPVs are more commonly found at anatomical sites that are exposed to sunlight, and a history of blistering sunburn is associated with prevalent and persistent cutaneous HPV infections [16,161,162,163]. In a case-control study, sunburn due to cutaneous sensitivity to sunlight exposure was associated with a higher seroprevalence for beta-HPV types [164]. There is also limited prior evidence suggesting a role of UVR in other animal models for papillomavirus infection. Early studies investigating UVR-induced tumors in hairless *HRA/Skh* (Short-ear, hairy and naked) mice [165] led to the identification of novel a papillomavirus, which, based upon Southern hybridization, shared homology with MnPV1 DNA, as well as with HPV11, -13, -16 and -18 DNA [166]. The viral genome, however, was not isolated or sequenced. Further, studies showed that cell-free extracts containing this MnPV1-like viral DNA enhanced UVR-induced tumorigenesis [167] suggesting that perhaps there is some relation between UVR and PV pathogenesis. The most convincing evidence supporting the role of UVR in animal infection models comes from studies with the cottontail rabbit papillomavirus (CRPV) where it was found that papillomas were induced in a subset of cottontail rabbits that were latently infected with CRPV when the animals were exposed UVR [168]. Furthermore, UVR has been identified as a cofactor in causing skin cancers in transgenic mouse models for several beta-HPVs (e.g., HPV8 [169], HPV20 [170], HPV27 [170], and HPV38 [171]).

In our studies, we made the novel observation that when FVB/NJ mice were infected with MmuPV1 and exposed to the UVB (Ultraviolet B) spectra of UVR, greater than 50% of infected sites developed papillomas, some of which persisted while the others completely regressed. UVB also induced MmuPV1-dependent papillomatosis in other strains of immunocompetent mice such as BALB/c and C57/BL6, but at higher doses of UVB. Histopathological analysis of the papillomas in the MmuPV1/UVB infection model also indicated progression to squamous cell carcinoma. While the malignant potential of MmuPV1 had been suggested in nude mice, this was the first evidence demonstrating that MmuPV1 can cause cancer in immunocompetent strains of mice. The most significant observation of this study was the finding that there was a strong correlation between long-term immunosuppression induced by UVB and MmuPV1-dependent pathogenesis. This correlation supports the hypothesis that UVB-induced immunosuppression can help to drive papillomavirus-induced disease. Epidemiological data from human studies indicates that anatomical sites on individuals that are exposed to sunlight have increased susceptibility to HPV-induced warts. However, in this mouse study, the effects of UVB were found to be systemic i.e., sites of infection did not have to be exposed to UVB irradiation. Further studies are needed to assess whether a local effect of UVB on MmuPV1 can be identified in mice.

UVR has been shown to enhance pathogenesis of cutaneous (e.g., herpes simplex virus-type 1 or HSV-1), as well as non-cutaneous based viruses (e.g., murine leukemia virus, influenza virus, and reovirus) [172]. The effects of UVR on viral pathogenesis can be indirect and/or direct. The indirect effects of UVR involve the modulation of host immunity and have been extensively reviewed elsewhere [173,174,175,176,177,178,179,180]. UVB plays a key role in initiating and mediating immunosuppression [181,182], and its effects can be long lasting [183,184,185]. UVR-dependent immunosuppression is initiated when chromophores (DNA, Urocanic acid) that are present in the skin first encounter UVR photons [186,187,188] and is largely T-cell mediated. UVB can inhibit the development of memory T-cells, as well as cause an overall reduction in T-cell subpopulations in the skin [189]. UVR causes the induction of immunosuppressive T-cell subpopulations (e.g., T_regs_) [190,191] and alters overall host cytokines from a pro-inflammatory Th1 to the immunosuppressive Th2 profile [192,193]. Recent studies have also implicated resident skin immune cells (e.g., langerhan cells, monocytes, macrophages) and keratinocytes in the process of UVB-induced immunosuppression [174,180]. While the effects of UVR on the host warrant further investigation, the observation that UVR can suppress immune responses to infectious microorganisms leads to the hypothesis that exposure to UVR can enhance susceptibility to microbial infections, particularly viruses, and/or worsen the state of infectious diseases [194,195]. Our data with MmuPV1 supports this hypothesis. Mechanisms underlying UVR induced-immunosuppression in the MmuPV1 pathogenesis model remain to be determined.

UVR can directly impact viruses and their host cells by causing mutations due to UVRs ability to form photo-adducts [196]. Studies performed in the context of HSV-1 and parvovirus support this hypothesis [197,198]. UVR may also lead to the formation of DNA-protein cross-links, the impacts of which are unknown [199]. In addition, UVR has been shown to enhance the frequency of viral recombination in both adenoviruses and HSV [200,201], which can potentially lead to more pathogenic variants of the viruses. UVR may directly induce viral transcription by acting on UVR response elements that are present in the promoters of certain viruses (e.g., HIV, HSV-1) [202,203]. The direct effects of UVR on papillomavirus biology remain to be understood. However, there is precedence to test roles of PV genes in modulating cellular responses to UVB. For example, beta HPVs (HPV5 and HPV8) E6 oncoproteins cause degradation of the pro-apoptotic protein Bak thereby inhibiting apoptosis [204,205]; on the other hand, HPV77 E6 oncoprotein has been shown to deregulate p53-dependent transactivation of proapoptotic genes upon UVB irradiation [206]. The physiological relevance of these properties, if any, can be assessed by means of rodent models, particularly, MmuPV1, as MmuPV1-E6 shares several properties in common with beta-HPVs (see below).

In the case of the HSV family of viruses, latent virus is reactivated by exposure to a variety of environmental or physiologic stimuli, including UVR radiation exposure. In our MmuPV1-UVR infection model we observed that a single exposure to UVB for as long as 14 days post-infection led to papillomatosis at sites that were infected with MmuPV1 [116]. This indicates either that the virus is stably retained at the site of infection or that latent infection arises that is then activated by UVB-induced immunosuppression. In support of the latter hypothesis, Doorbar and colleagues have observed that rabbit oral papillomavirus-induced lesions that regress can be reactivated upon drug-induced immunosuppression [207]. It remains to be determined whether in the MmuPV1 infection model latency arises, and, if so, the nature of this latency.

### 3.3. Other Rodent Papillomaviruses

Besides MnPV1 and MmuPV1, MmiPV1 is another rodent papillomavirus that has been identified to infect the European harvest mouse belonging to the *Muridae* phyla of rodents. MmiPV1 was isolated from lesions arising in the European harvest mice housed in a regional zoo in Chicago. Initial efforts in characterizing MmiPV1 suggest that it is a cutaneous papillomavirus as no lesions were identified in any internal organs. The virus was found to be defective in transforming 3T3 and C127 cells [53,208]. The complete genome of MmiPV1 was sequenced fairly recently [52], making it possible to understand further the biology of this virus. Recently, three novel papillomaviruses that can infect rat species (RnPV1-RnPV3) have been isolated [47]. These can be potentially useful models to study papillomavirus infections as rat models can also be studied in the laboratory setting.

## 4. Insights into Vaccine Development

There are few studies that have evaluated therapeutic approaches to treat MnPV1-induced papillomas. A modest response was seen in MnPV1-induced spontaneous papillomas in *M. coucha* treated with the immune response modifier imiquimod [209]. Recently, it has been shown that vaccinating *M. coucha* with an L1-based virus like particle (VLP)-based vaccine can prevent MnPV1-induced lesions (benign as well as malignant) in both naturally infected strains of *M. coucha* as well as experimentally infected naïve *M. coucha* animals. The authors also observed long-lasting humoral immune responses following vaccination in animals that had been chronically immunosuppressed via the administration of cyclosporine A. The results of this study have valuable implications in the field of vaccine development, as the authors demonstrated that VLP-based vaccines could effectively prevent the appearance of skin tumors, even in animals that are already infected or undergo systemic immunosuppression [88]. Vaccination of immunosuppressed individuals with HPV-vaccines has produced modest results due to low seroconversion but recommendations for three doses of the vaccine have been made, which merit further research [210]. Little is known about the similarities of the *M. coucha* immune system with the human immune system; therefore, the MmuPV1-infection model is potentially a more relevant animal model, given the extensive knowledge of the *Mus musculus* immune system and its relevance to humans. In this context, recently the Roden group has tested a naked-DNA vaccine expressing human calreticulin (hCRT) fused in frame to MmuPV1 E6 (mE6) and mE7 early proteins, and residues 11 to 200 of the late protein L2 (hCRTmE6/mE7/mL2) in the MmuPV1-infection model established in SKH1-e (hairless mice), in which a low percentage of animals are susceptible to MmuPV1 infections [211]. In this study the authors found that persistent papillomas arising due to MmuPV1 infections disappeared within 2 months after final vaccination, and the disease did not recur when CD3 T-cells of these animals were depleted.

## 5. Transcript Maps of Rodent Papillomaviruses

**Figure 4:**
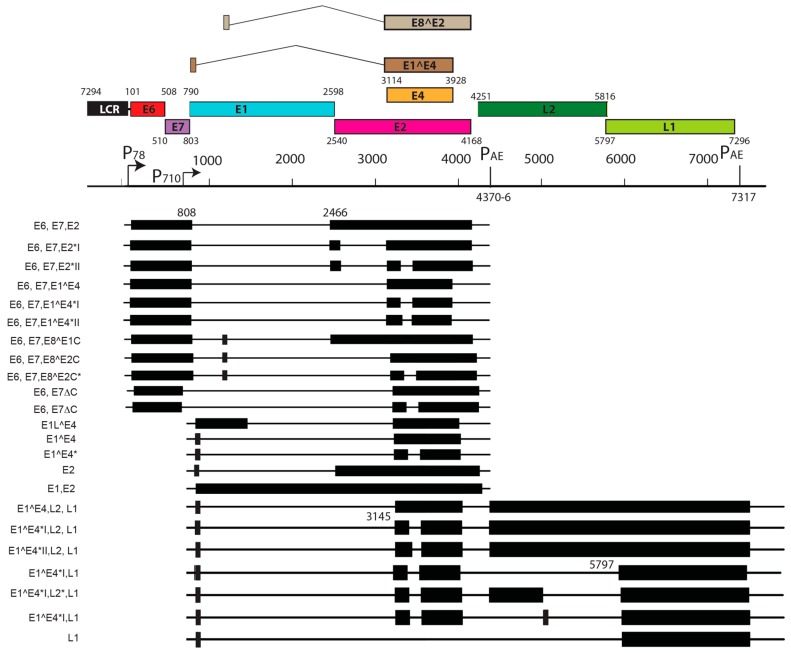
Transcription map of MnPV1. This figure has been adapted and modified from [97]. The full transcription map for MnPV in productive lesions was assembled from PCR, RACE, and RNA-seq data. At the top, the genome organization of MnPV is presented for better understanding; numbers indicate the position of ORF starts and ends. Mapped TSSs 78 and 710 and pA cleavage sites 4370-6 and 7317 are depicted. For transcripts, exons are represented by black boxes (when coding for a particular ORF) while introns are marked as thin solid lines. Positions of the splice junctions in each transcript are given by numbers at the top.

**Figure 5:**
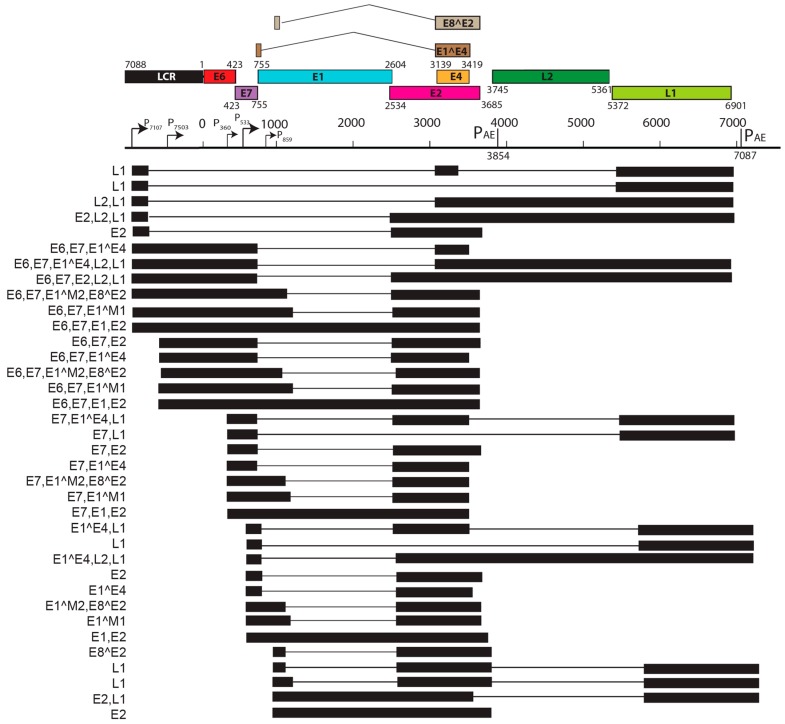
Transcription map of MmuPV1. This figure has been modified and adapted from Xue, et al. (2017) (Manuscript accepted [102]) The bracket line in the middle of the panel represents a linear form of the virus genome for better presentation of head-to-tail junction, promoters (arrows), and polyadenylation cleavage sites, early pA CS 3864 and late pA CS7063. The open reading frames (ORFs) are diagramed above the bracket line as colored boxes, and the numbers above each ORF are nucleotide positions of the first nucleotide of the start codon and the last nucleotide of the stop codons in the MmuPV1 genome. LCR indicates a long control region. Below the bracket line are the RNA species derived from alternative promoter usage and alternative RNA splicing. Exons (black boxes) and introns (thin lines) are illustrated for each species of the RNA, with the mapped splice site positions numbered by nucleotide position in the virus genome and coding potentials are shown on the left.

Viral transcript maps have been analyzed for two rodent PVs, MnPV1 [97], and MmuPV1 [102] (Figure 4 and Figure 5). Both of these transcript maps were generated using cutting edge RNA sequencing technologies in combination with classical techniques to determine mRNA coding regions leading to the generation of comprehensive transcription maps that will be very valuable to the field of papillomavirus biology. The MmuPV1 transcript map also made use of PacBio Iso-seq in combination with RNA seq to generate a very thorough transcript map. The salient features of the MnPV1 and MmuPV1 transcript maps are summarized in Table 5. Similar to other PVs, both MnPV1 and MmuPV1 have a complex array of mono- and poly-cistronic transcripts arising from several promoters and multiple splice sites that are empowering these compact viral genomes to express multiple genes. In the case of MnPV1, similar to several high-risk HPVs (e.g., HPV types 16, 18 and 31), the early promoter (P_78_) is located upstream of E6 within the long control region (LCR) [212,213,214], leading to transcripts that can drive expression of both E6 and E7 gene products. A TATA box was identified upstream of the transcriptional start site (TSS) for this promoter. In contrast, similar to cutaneous HPVs and certain low-risk mucosotropic HPVs [215,216], MmuPV1 makes use of two separate early promoters for expression of viral E6 (P_7503_) and E7 (P_360_) with P_7503_ being a stronger promoter. A third early promoter (P_859_) was also identified for MmuPV1 and is most likely a weak promoter that is responsible for driving expression of *E2* and/or *E8^E2*. High-risk HPVs usually express their late genes mainly from a differentiation-dependent TATA-less late promoter located within the *E7* ORF. Similar to these HPVs, a strong late promoter was identified within the *E7* ORF (P_710_) of MnPV1 with a TATA-like sequence upstream of the TSS [97]. Interestingly, in the case of MmuPV1, two late promoters, P_7107_ (within the LCR region downstream of L1 ORF) and P_533_ (within the *E7* ORF), were identified. Of these, P_7107_ has a TATA-like box upstream of its TSS, and the P_533_ bears a TATA box upstream of its TSS. Utilization of a late promoter in the LCR region for the expression of viral late genes *L1* and *L2* is a characteristic feature for BPV1, CRPV, and some skin-tropic HPVs, such as HPV1 and HPV5 [215,217,218,219]. By means of cutting edge technologies that were employed in constructing the MnPV1 and MmuPV1 transcript maps, several novel splice junctions, as well as transcripts, have been identified. In transcription maps of both MnPV1 and MmuPV1 the *E8^E2* splice isoform was detected. The E8^E2 protein is encoded by a spliced message containing a short exon from the *E8* ORF spliced to the major splice acceptor in the middle of the *E2* ORF [76]. Transcripts encoding shorter E2 forms have been mapped in BPV1 and have been shown to repress E2-dependent transactivation of BPV1 E2-dependent enhancer [220] and to inhibit viral DNA replication [221]. These E2 repressors have also been mapped in CRPV1, HPV11, HPV16, and HPV31 [222,223,224,225]. Recently, in monolayer keratinocytes stably harboring HPV16, it was shown that E8^E2 represses the productive replication of HPV16 and knocking-out *E8^E2* increased HPV16 copy numbers [226]. The use of rodent models provides us with the opportunity to understand the pathogenic relevance of this repressor protein in animal species. Besides *E8^E2*, in MnPV1 potential E2 repressors in the form of E2*I and E2*II were also identified. In the case of MmuPV1, splice isoforms E1^M1 and E1^M2 have been identified. These are predicted to be similar to E1Ma and E1M in HPV11, which encode fusion proteins that can strongly repress both E2-dependent and independent enhancer/promoter activities of HPV11, which is a low-risk genital HPV [223]. While the functional significance of these several isoforms remains to be elucidated for rodent PVs, broadly speaking we can say that, based on transcriptional analysis alone, MnPV1 appears to be more similar to high-risk mucosotropic HPVs and MmuPV1 appears to be more similar to high-risk cutaneous HPVs.

## 6. Roles of Rodent PV’s E6 and E7 Proteins

Preliminary studies using MnPV1 E6 transgenic mice suggest an oncogenic role in MnPV1 [95]. In the context of MmuPV1, *E6* is necessary for virus-induced papillomas in nude mice [227]. This suggests that *E6* is potentially critical in viral pathogenesis for rodent PVs, and its function merits further investigation. E6 proteins are approximately 150 amino acids in length, containing two CX2C-X29-CX2C zinc-like fingers, while being flanked by short amino (N) and carboxy (C) terminal domains of variable lengths [80]. Like most PVs, rodent PV E6 proteins also have these zinc-like finger domains (Supplementary Figure S1). Multiple cellular proteins have been identified to associate with E6 proteins. The primary interaction seen with mucosal and cutaneous HPV’s E6 and BPV1 E6 is their binding to an alpha-helical acidic LXXLL peptide motif present in multiple cellular target proteins. This interaction has been validated by solving the crystal structures of HPV16 E6 bound to E6AP (E6 association protein) peptide, and BPV1 E6 bound to paxillin peptide [228]. Some well-known binding partners of papillomaviruses containing the LXXLL domain include, paxillin, E6AP, Mastermind-like 1 (MAML-1), and interferon regulatory factor-3 (IRF3) [229,230,231,232,233,234,235]. Among these, the interaction of HPV16 E6 and HPV18 E6 with E6AP is perhaps the most widely studied interaction due to the ability of these E6 proteins, together with E6AP, a ubiquitin ligase, to form a complex with the tumor suppressor p53 [230] targeting the latter for proteasome-mediated degradation [236]. Two groups identified Mastermind-like 1 (MAML1), of the Notch transcription complex, as a cellular interacting protein of the E6 proteins encoded by BPV1 and the beta-HPVs [234,237]. MAML1 is a core component of the transcriptional activation complex that mediates the effects of the canonical Notch signaling pathway [238]. More recent studies of HPV8 E6 (a β-HPV) demonstrated that Notch activation was subverted by suppressing the function of MAML1 during keratinocyte differentiation [235]. MmuPV1 E6 shares with the skin cancer-associated HPV8 E6 protein the capacity to inhibit Notch signaling by binding to MAML1, and MmuPV1-E6 mutants that are defective in MAML1 binding are defective in causing MmuPV1-induced papillomas [227]. Interestingly, most high-risk mucosotropic HPV E6s bind to E6AP, whereas most high-risk cutaneous HPV E6s bind to MAML1 [231]. The significance of this difference in the preference of different E6 proteins to bind different LXXLL motif-containing cellular proteins is yet to be determined, however, rodent PV models, specifically MmuPV1, can now be used to understand the significance of this association in pathogenesis.

Amino acid sequence comparisons of rodent PV *E6*s suggest other potentially interesting putative roles as well. For example, MmuPV1 *E6* contains an LXCXE motif that is implicated in binding to retinoblastoma protein (RB). This motif is more commonly found in the E7 proteins in other papillomaviruses [59]. In the case of MnPV1, the LXCXE domain is present in both E6 as well as E7 (Supplemental Figures S1 and S2). LXCXE motifs in E6 proteins were also observed in *Phocoena spinipinnis* papillomavirus type 1 (PsPV1) and bottlenose dolphin PV types 1, 2, and 3 (TtPV1–3), which do not have identifiable *E7* ORFs. While LXCXE-independent interactions of E7 with RB have been reported for canine papillomaviruses [239], deeper analysis of such interactions, if any, is required in the context of rodent PVs. A unique characteristic of the high-risk HPV E6 oncoproteins is the presence of a PDZ (PSD-95/DLG/ZO-1) binding site motif that is designated by the amino-acid sequence XS/TXV on the extreme C-terminus. This motif is absent from all of the so-called low-risk HPV E6 oncoproteins [240]. None of the rodent papillomaviruses possess PDZ binding motifs on the extreme C-terminus of either *E6* or *E7* genes; however, putative PDZ binding site motifs can be found elsewhere in both *E6* and *E7*s of several rodent PVs (Supplemental Figures S1 and S2).

The other well-studied HPV oncogene is *E7*. The ability of *E7* in high-risk mucosal papillomaviruses to cause cancer is often attributed to the ability of E7 to interact with UBR4 and cullin family ubiquitin ligases and to bind to the RB family of proteins (p105, p107, and p130) that regulate E2 factor (E2F) family transcription factors [241]. While high-risk *E7s* are oncogenic, *E7* from low-risk viruses (HPV types 6, 11) are weakly oncogenic and have cooperative activity when they are co-expressed with additional oncogenes from the high-risk viruses [242]. A hallmark feature of high risk HPV E7 proteins is their ability to bind the tumor suppressor RB through their LXCXE motif, [243]; however, this is not the case for E7 proteins encoded by other papillomaviruses. For example, BPV1 *E7* does not contain a LXCXE motif. In fact, the E7 proteins that are encoded by all of the animal papillomaviruses that cause fibropapillomas lack this motif. This is also true for avian papillomaviruses [244]. Interestingly, some papillomaviruses have no *E7* gene at all (isolates from domestic pigs, SsPV, polar bear UmPV, and porpoises) [245,246,247]. Furthermore, many RB-independent biological activities of E7 have also been reported [248]. One such activity is the ability of high-risk E7s to bind and degrade the cellular non-receptor protein tyrosine phosphatase (PTPN14). Recently, it has been demonstrated that PTPN14 degradation correlates with the retinoblastoma-independent transforming activity of high-risk HPV E7. Interestingly, immunoprecipitation-tandem mass spectrometry (IP-MS/MS) experiments have determined that the MmuPV1 E7 does bind to PTPN14, suggesting that this rodent model is a valuable model for PV carcinogenesis [249]. Interaction partners of E6 and E7 of other rodent PVs are yet to be determined and provide a plethora of opportunities.

## 7. Conclusions

In conclusion, rodent models of papillomavirus infection are a fledgling area of research that merit further investigation. The progress made in rodent PV research, particularly the MmuPV1 infection model, provides us with great opportunities to understand the biology of papilloma virus. These models can help us understand multiple unanswered questions about papilloma viruses particularly in the context of virus tropism and host immunity, as these are easily tractable infection models. For MmuPV1, we have a wide array of genetic tools that are available to answer several of these questions.

## Figures and Tables

**Table 1:** List of Rodent papillomaviruses.

S. No.	PV	Genome Size bp	PAVE ^1^ Name	Host Common Name	Genus	Reference
1	AsPV1	7589	*Apodemus sylvaticus* Papillomavirus 1	Long-tailed field mouse	π	[47]
2	CcanPV1	7435	*Castor canadensis* Papillomavirus 1	North-American beaver	*Dyosigma*	[48]
3	EdPV1	7428	*Erethizon dorsatum* Papillomavirus 1	North-American porcupine	σ	[49]
4	MaPV1	7647	*Mesocricetus auratus* Papillomavirus 1	Syrian golden hamster	π	[50]
5	McPV2	7522	*Mastomys coucha* Papillomavirus 2	Southern multimammate rat	π	[51]
6	MmiPV1	7393	*Micromys minutus* Papillomavirus 1	European harvest mouse	π	[52,53]
7	MmuPV1 ^2^	7501	*Mus musculus* Papillomavirus 1	House mouse	π	[54,55]
8	MnPV1	7687	*Mastomys natalensis* Papillomavirus 1	Southern multimammate rat	ι	[56,57]
9	PsuPV1	7630	*Phodopus sungorus* Papillomavirus 1	Siberian hamster	π	[58]
10	RnPV1	7378	*Rattus norvegicus* Papillomavirus 1	Norwegian rat	π	[47]
11	RnPV2	7724	*Rattus norvegicus* Papillomavirus 2	Norwegian rat	ι	
12	RnPV3	7707	*Rattus norvegicus* Papillomavirus 3	Norwegian rat	ι	

^1^ Papillomavirus Episteme: https://pave.niaid.nih.gov/; ^2^ Mouse papillomavirus was originally designated as MusPV1 [54,59].

**Table 2:** List of immunodeficient mice found to be susceptible to MmuPV1 skin infections.

S. No.	Deficiency	Strain-*Mutation*	Reference
1	Lack T cells	B6.Cg-*Foxn1^nu^*/*J*	[55,99]
2		BALB/c-*Foxn1^nu^*	[116]
3		Hsd:NU-*Foxn1^nu^*	[119]
4		NCr nu/nu	[98]
5		NMRI-*Foxn1^nu^*/*Foxn1^nu^*	[54]
6		NU-*Foxn1^nu^*/*Foxn1^nu^*	[103]
7	Lack T & B cells	B6.129S7-*Rag1^tm1Mom^*/*J*	[99,120]
8		B6.CB17-*Prkdc^scid^*/*SzJ*	[99]
9		NCI SCID/Ncr	[120]
10		SHO-*Prkdc^scid^Hr^hr^*	[118]

**Table 3:** List of immunodeficient mice found to be resistant to MmuPV1 skin infections.

S. No.	Deficiency	Strain/Mutation	Reference
1	Lack T & B cells	NOD.CB17-*Prkdc^scid^*/*SzJ*	[99]
2	Lack B cells	B6.129S2-Ighm^tm1Cgn^/J	[99]
3	Lack CD4^+^ T cells	B6.129S2-Cd4^tm1Mak^/J	[99]
4	Lack CD8^+^ T cells	B6.129S2-Cd8^tm1Mak^/J	[99]
5	Lack T helper cells/CD40 ligand K	B6.129S2-Cd40lg^tm1Imx^/J	[120]
6	Lack NK (CD1d) cells	B6.129S6-Del (3Cd1d2-Cd1d1)^1Sbp^/J	[126]
7	Decreased Anti-viral innate immunity, lack Type I Interferon	IFNAR KO	[120]

**Table 4:** List of immunocompetent mice found to be susceptible to MmuPV1 after treatment with immunosuppressants.

S. No.	Strain	Intervention	Reference
1	Cr:ORL SENCAR	Cyclosporine	[126]
2	FVB/NCr		
3	BALB/cAnNCr		
4	A/JCr		
5	Cr:ORL SENCAR	Anti-CD3	[126]
6		Anti-CD4	
7		Anti-CD8	
8	C57BL/6	Anti-CD3	[120,126]
9		Anti-CD4+Anti-CD8 *	
10	BALB/c	Anti-CD3 *	[120]

* Anti-CD4 or Anti-CD8 alone did not lead to warts.

**Table 5:** Key features of MnPV1 and MmuPV1 transcript maps.

Feature	MnPV1	MmuPV1
Source	Skin papillomas from *M. Natalensis* colonized with MnPV1	Skin papillomas from immunodeficient mice infected with MmuPV1
Early Promoters	Single promoter (P_78_) for *E6* and *E7*	Three promoters: P_7503_ for E6, P_360_ for E7, P_7503_ for *E2* and/or *E8^E2*
Late Promoters	Single late promoter (within *E7* ORF)	Two late promoters (within LCR and *E7* ORF)
Notable splice isoforms	E8^E2, E2*I, E2*II	E1^M1, E1^M2, E8^E2

## Supplementary Materials

The following are available online at www.mdpi.com/1999-4915/9/12/362/s1, Figure S1: E6 amino acids of rodent papillomaviruses from Table 1 were aligned in JalView (software version: 2.9.0b2, The Barton Group, University of Dundee, Scotland, UK) [250] using the ClustalX algorithm. Two zinc-like finger domains with consensus sequence CX2C-X29-CX2C are highlighted. Numerous PDZ binding site motifs (XS/TXV, magenta box) were seen throughout the E6 sequence. The Rb binding motif (LXCXE, white box) was seen in MmuPV1 and MnPV1. Figure S2: E7 amino acids of Rodent Papillomaviruses from Table 1 were aligned in JalView [250] using the ClustalX algorithm. One zinc-like finger domain with consensus sequence CX2C-X29-CX2C was seen near the C-terminus. Numerous PDZ binding site motifs (XS/TXV, magenta box) were seen throughout the E7 amino acid sequence of rodent PVs. Rb binding motif (LXCXE, white box) was seen in MnPV1, RnPV2 and RnPV3.
