# Supplementary material for: Rodent Papillomaviruses
Source: Viruses. 2017 Nov 27;9(12):362. doi: 10.3390/v9120362 (PMC5744137; doi:10.3390/v9120362)
Supplement: Supplementary file 1 [file viruses-09-00362-s001.pdf]

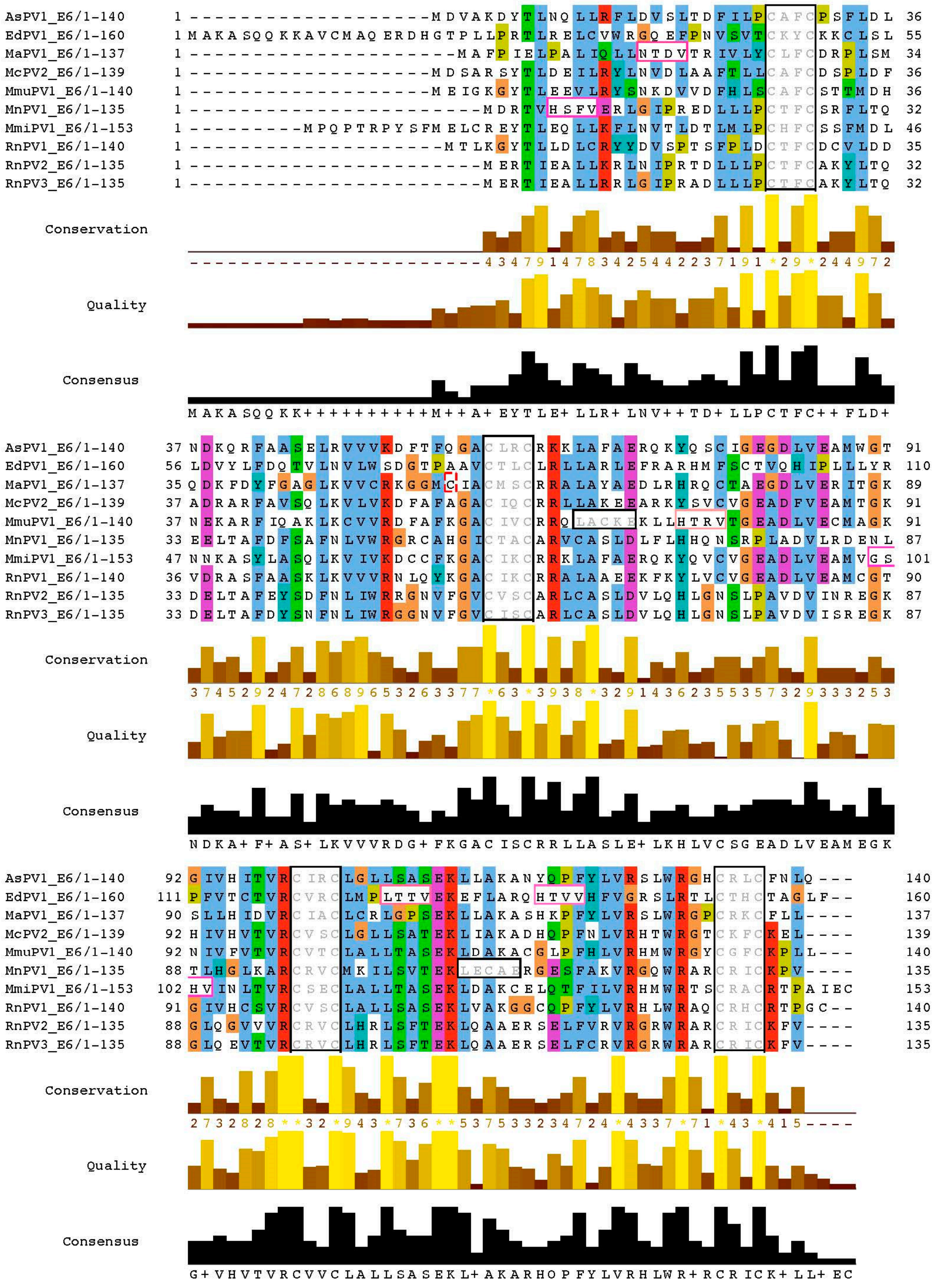

Figure S1: E6 amino acids of rodent papillomaviruses from Table 1 were aligned in JalView [252] using the ClustalX algorithm. Two zinc-like finger domains with consensus sequence CX2C-X29-CX2C are highlighted. Numerous PDZ binding site motifs (XS/TXV, magenta box) were seen throughout the E6 sequence. The Rb binding motif (LXCXE, white box) was seen in MmuPV1 and MnPV1.

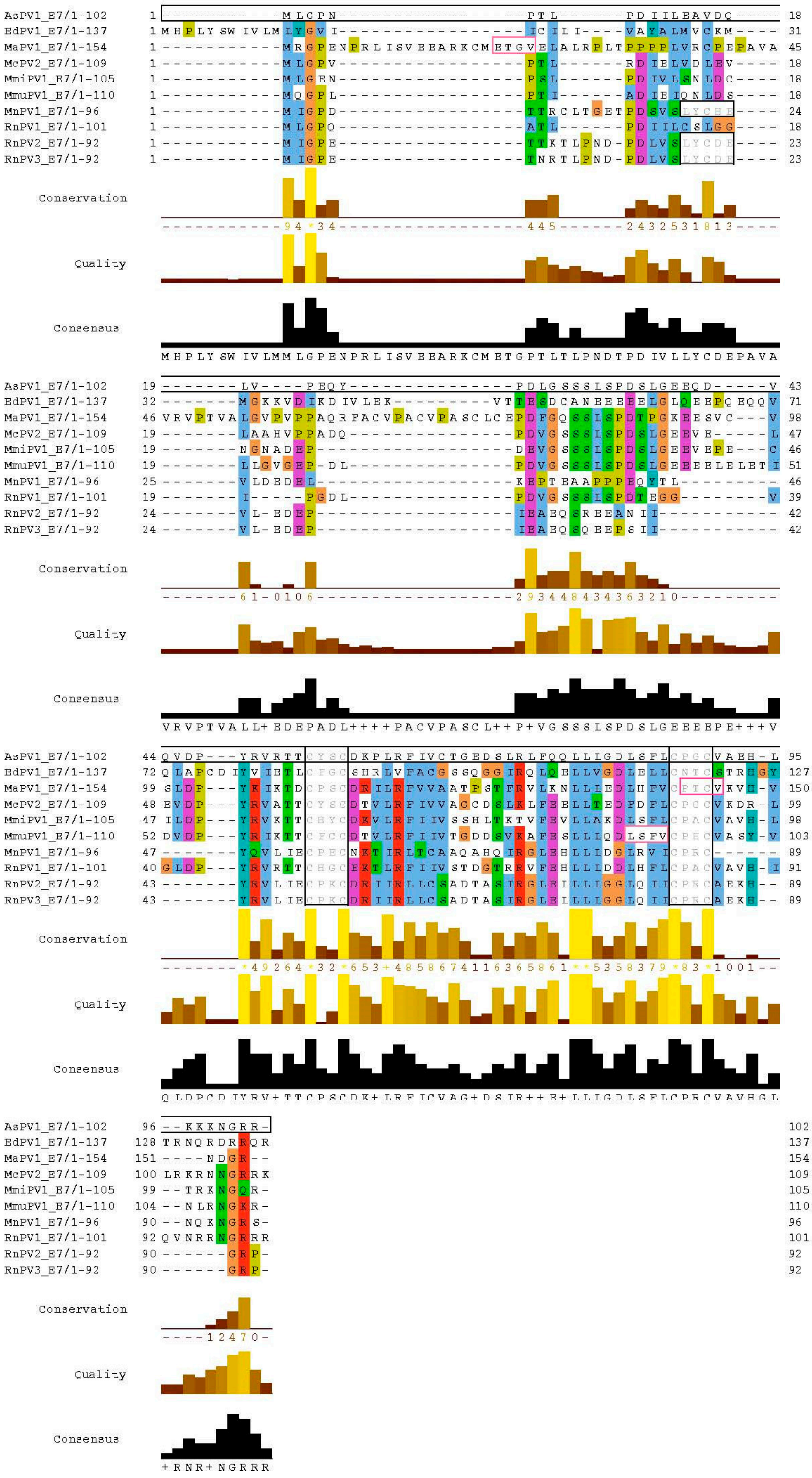

Figure S2: E7 amino acids of Rodent Papillomaviruses from Table 1 were aligned in JalView [252] using the ClustalX algorithm. One zinc-like finger domain with consensus sequence CX2C-X29-CX2C was seen near the C-terminus. Numerous PDZ binding site motifs (XS/TXV, magenta box) were seen throughout the E7 amino acid sequence of rodent PVs. Rb binding motif (LXCXE, white box) was seen in MnPV1, RnPV2 and RnPV3.
